# Single-Shot Direct Transmission Terahertz Imaging Based on Intense Broadband Terahertz Radiation

**DOI:** 10.3390/s24134160

**Published:** 2024-06-26

**Authors:** Zhang Yue, Xiaoyu Peng, Guangyuan Li, Yilei Zhou, Yezi Pu, Yuhui Zhang

**Affiliations:** 1 Center of Quantum Information Technology, Chongqing Institute of Green and Intelligent Technology, Chinese Academy of Sciences, Chongqing 400714, China; yuezhang21@mails.ucas.ac.cn (Z.Y.); liguangyuan@cigit.ac.cn (G.L.); zhouyilei@cigit.ac.cn (Y.Z.); puyezi21@mails.ucas.ac.cn (Y.P.); zhangyuhui221@mails.ucas.ac.cn (Y.Z.); 2 Chongqing School, University of Chinese Academy of Sciences, Chongqing 400714, China

**Keywords:** fast THz imaging, THz transmission imaging, intense broadband THz radiation, multi-scale retinex

## Abstract

There are numerous applications of terahertz (THz) imaging in many fields. However, current THz imaging is generally based on scanning technique due to the limited intensity of the THz sources. Thus, it takes a long time to obtain a frame image of the target and cannot meet the requirement of fast THz imaging. Here, we demonstrate a single-shot direct THz imaging strategy based on a broadband intense THz source with a frequency range of 0.1~23 THz and a THz camera with a frequency response range of 1~7 THz. This THz source was generated from the laser–plasma interaction, with its central frequency at ~12 THz. The frame rate of this imaging system was 8.5 frames per second. The imaging resolution reached 146.2 μm. With this imaging system, a single-shot THz image for a target object with a size of more than 7 cm was routinely obtained, showing a potential application for fast THz imaging. Furthermore, we proposed and tested an image enhancement algorithm based on an improved dark channel prior (DCP) theory and multi-scale retinex (MSR) theory to optimize the image brightness, contrast, entropy and peak signal-to-noise ratio (PSNR).

## 1. Introduction

A THz wave generally refers to an electromagnetic wave with frequencies ranging from 0.1 to 10 THz or wavelengths ranging from 3 to 0.03 mm, falling in the gap between microwave and infrared. This band occupies a special position in the electromagnetic spectrum [1]. Because of their special properties, such as excellent penetrability of non-metallic materials [2], high resolution [3] and sensitive interaction with biological macromolecules [4], THz waves have been used in various fields. At present, the main research on THz technology focuses on THz time-domain spectroscopy and THz imaging technology. After the rapid development of THz technology in recent decades, THz imaging has become more mature. It has been developed for different scenarios, such as THz time-domain spectral imaging [5,6], THz near-field imaging [7,8,9,10,11,12,13,14], THz tomography imaging [12,13,14], THz digital holographic imaging [15,16], THz compressed sensing imaging [17,18,19,20], etc. These THz imaging techniques have been widely used or have shown potential applications in security [21], medical science [22], industrial non-destructive testing [23] and other fields [24,25].

Most current THz imaging techniques mainly use scanning due to the weak THz sources. In these schemes, the THz beam is focused on the target object, transmitting or being reflected from the occlusive object. The THz image of the target object can be obtained by scanning the THz focus over the target object generally by moving the target object. For example, Banerjee et al. [26] built a THz scanning imaging system at 0.6 THz, with a spatial resolution of 1 mm. They spent 1.7 min scanning one frame for a total of 40 pixels × 40 pixels. Zhao et al. [27] constructed a transmission continuous THz scanning imaging system with imaging resolution of 260 μm. In the abovementioned scanning systems, the imaging process is too slow to be used in many applications [28,29], so it is desirable to develop fast THz imaging techniques. In the early THz real-time imaging systems based on array receivers, THz sources based on molecular lasers, quantum cascade lasers and free electron lasers [30,31,32,33] were used. Yasuda et al. [34] built a transmission THz real-time imaging system using complementary metal-oxide-semiconductor (CMOS) cameras, with an imaging resolution of 1 mm. Han et al. [35] built a THz real-time imaging system based on InGaAs Schottky diode array detectors. The acquisition speed reaches 20 frames per second and the maximum scanning area is 25 × 25 mm^2^. The imaging resolution is 0.5 mm. Even though the above conceptual fast THz imaging techniques have shown some potential applications [36,37,38], there are still challenges in real applications due to the small imaging range, insufficient resolution, weak brightness and poor image contrast that are limited by the THz sources.

In this work, we constructed a single-shot fast THz imaging system based on an intense broadband laser-plasma THz source and a THz camera. With this system, a direct transmission THz image of a hidden target object with a size of ~7 cm and a frame rate of 8.5 frames per second (fps) can be realized. Based on the characteristics of the imaging results of the system, we also propose an image enhancement algorithm based on the improved dark channel prior theory and the improved multi-scale retinex theory to improve the image quality.

## 2. Experimental Setup and Imaging Results

The intense broadband THz source used in this imaging experiment was generated from laser–plasma interaction, pumped by a femtosecond laser amplification system Spitfire Ace PA produced by the Spectral-Physics in California, USA. This laser system delivers laser pulses with a pulse duration of 40 fs and laser energy of 4.5 mJ at a repetition rate of 1 kHz. The central wavelength of this laser is ~800 nm. The diameter of the output laser beam is ~12 mm measured at the intensity of 1/e^2^ of the laser beam.

**Figure 1:**
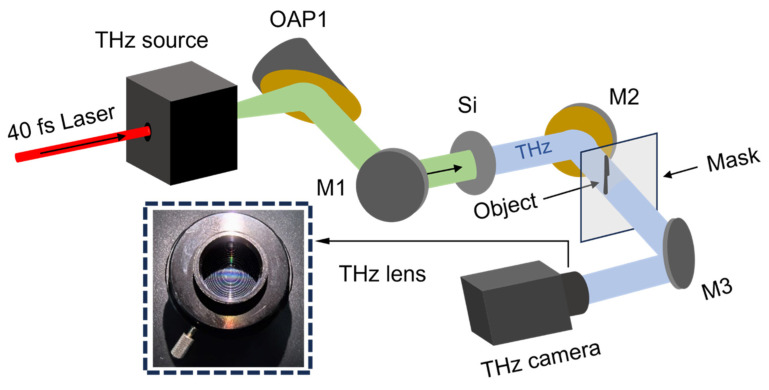
Optical layout of the single-shot fast THz imaging system.

The experiment layout is shown in Figure 1. An intense broadband THz radiation beam from laser–plasma interaction, similar to that in the work of Dey et al. [39], was collected and collimated by an off-axis golden protected parabolic mirror (76.2 mm in diameter), OAP1, then reflected by two gold-protected flat mirrors, M1 and M2, to the THz imaging area. A high resistance silicon wafer with resistivity >20,000 Ω · cm (as shown by Si in Figure 1) was inserted in the path between M1 and M2 to obtain a pure broadband THz wave by filtering the remnants of the pump laser beams and the white light as well as the infrared from the laser–plasma interactions. The pulse energy of the THz radiation reached 150 μJ. The diameter of the collimated intense broadband THz beam was 76.2 mm, thus a cross section of 45.58 cm^2^ of the THz beam was obtained for THz imaging. The frequencies of the THz wave fell in the range of 0.1~23 THz and the central frequency was ~12 THz. After being reflected by the mirror M2, the THz beam passed through the occluded objects (as shown by the object and the mask in Figure 1) and was collected by the third gold-protected flat mirrors, M3, and then reflected into a THz camera (IR/V-T0831C from NEC Corporation in Tokyo, Japan) for imaging. This THz camera from NEC Corporation had 320 × 240 pixels with a pixel size of 23.5 μm and a frequency response range of 1~7 THz [40,41], falling in the range of our intense broadband THz source (0.1~23 THz). Its equivalent noise power was <100 pW and its frame rate was 8.5 Hz. This THz camera was equipped with a focusing lens with a focal length of 28.2 mm and an F-number of 1.0. Fresnel lenses were used in the lens to compensate for color difference and reduce lens thickness. This Fresnel lens is produced by NEC Corporation in Tokyo, Japan.

**Figure 2:**
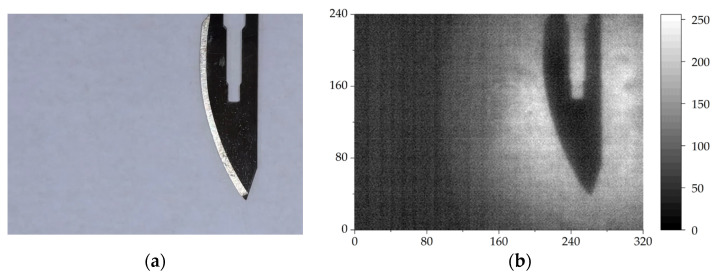
A hidden surgical blade in (**a**) and its single-shot THz image in (**b**).

To obtain a typical direct transmission THz image of a hidden target object, A4 paper, commonly used in daily life, was used as an opaque object covering. The thickness of a piece of A4 paper is 90 μm. In this experiment, a surgical blade was used as the imaging target object. Figure 2a shows the surgical blade, made of stainless steel with a bar hole.

By performing single-shot THz fast imaging on the hidden surgical blade covered by a piece of paper, a THz image of the blade was routinely obtained. Figure 2b shows a THz image of the hidden surgical blade. The THz imaging pattern clearly displays the contour and size of the surgical blade with details such as the central hole. According to the theory of optical diffraction limit, the image resolution of this imaging system can be expressed as

(1) $$\sigma =\frac{0.61\lambda }{NA},$$

here, *λ* is the wavelength of the THz source, and *NA* is the numerical aperture of the system. The resolution of the imaging system at the central frequency of ~12 THz of the THz waves is estimated to be 30.5 μm according to Equation (1) even though the pixel size of the THz camera was 23.5 μm. The calculated actual imaging resolution was approximately 146.2 μm based on the object size and the number of pixels in the object size in the imaging results.

**Figure 3:**
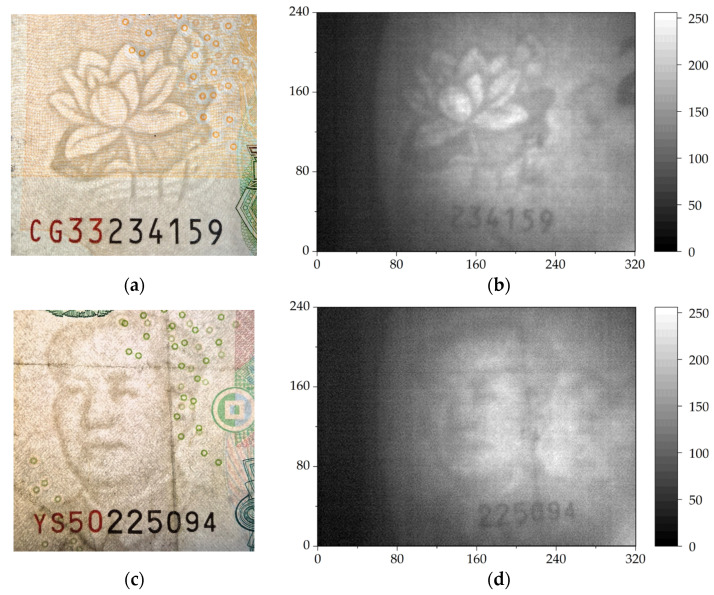
The backlit photos of the watermark patterns of the 20 yuan (**a**) and 50 yuan (**c**) banknotes and their THz transmission images (**b**,**d**) after being covered with a piece of paper.

To further demonstrate the practicality of the system, we performed THz transmission imaging on the watermarks on 20 yuan and 50 yuan banknotes (CNY), each covered by a piece of paper. We took backlit photos of the watermarks on the 20 yuan and 50 yuan banknotes for comparison, as shown in Figure 3a,c, respectively. The watermark on the 20 yuan is a lotus, and that on the 50 yuan is a portrait of Mao Zedong. The THz images are shown in Figure 3b,d. In these THz images, the watermarks are very clear, displaying the details of the patterns. Even the creases on the banknote in Figure 3c can be observed in the THz image shown as Figure 3d.

**Figure 4:**
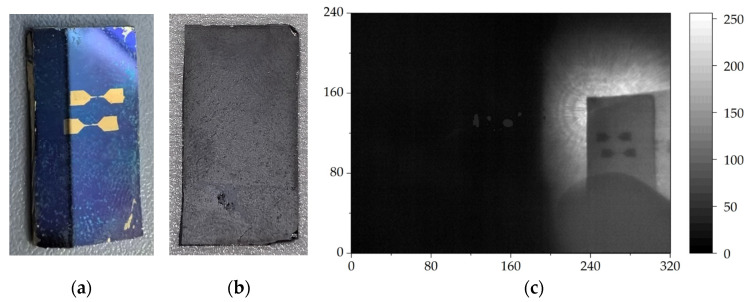
The frontside of a silicon wafer fabricated with two gold antennas on the surface (**a**) and the backside of this silicon wafer (**b**), with the THz transmission image of the backside of the silicon wafer (**c**).

To demonstrate further the transmission ability of the THz imaging system, we performed THz transmission imaging of a silicon wafer fabricated with two gold antennas on the front surface, as shown in Figure 4a. Figure 4b shows the backside of the silicon wafer with a thickness of 750 μm. The gold antennas had two triangles at both ends and thin lines connecting in the middle, resembling a dumbbell. The transmission THz image taken from the backside of the silicon wafer clearly identified the antennas and the structure, as shown in Figure 4c.

As the repetition rate of THz radiation is 1 kHz, which is dependent on the same rate of the laser system, real-time THz imaging with 24 fps could be realized in principle. However, the real imaging frame rate in our THz imaging system was limited by that of the THz camera, which was only 8.5 fps. Even so, our THz imaging setup was still a fast, direct transmission THz imaging system due to its single-shot imaging technique.

## 3. Image Processing

**Figure 5:**
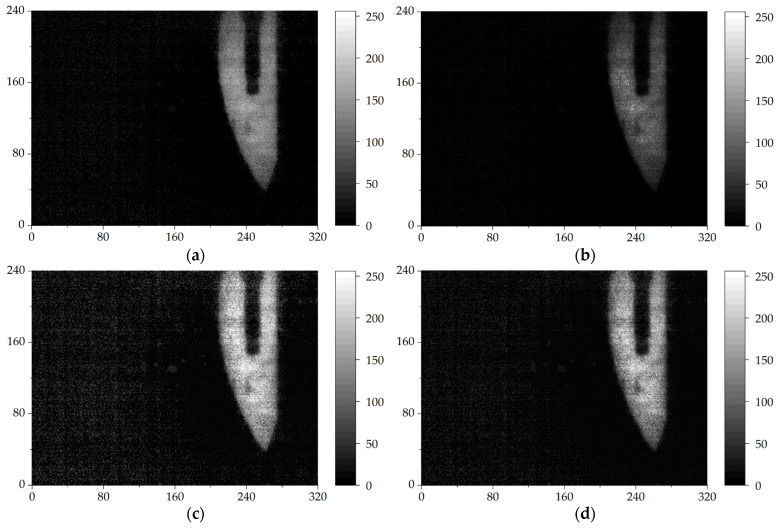
The processed THz images of the hidden surgical blade, showing subtraction of the background image (**a**) by enhancing the image with the dark channel prior algorithm (**b**), enhancing the image with the multi-scale retinex theory (**c**), and the improved image enhancement algorithm (**d**), respectively.

To improve the quality of the image, we first took the background image of the field of view without a target object, then subtracted the background image from the target object image. The subtracted image looked clearer, as shown in Figure 5a. The background information outside the target object disappeared and the object appeared more prominent. However, the edge of the blade became blurry and there was still some residual noise around the blade.

To further remove noise in the image, we developed an image enhancement algorithm based on an improved dark channel prior theory and an improved multi-scale retinex theory. There was a lot of foggy noise in the image, and we hoped to use image dehazing algorithms for image optimization.

The dark channel prior algorithm is a classic dehazing algorithm proposed by He et al. [42] in 2009. This method relies on atmospheric scattering models for image dehazing. They observed and summarized many foggy and non-foggy images to obtain mapping relationships. Based on the formation process of foggy images, they performed inverse operations to restore clear images. Despite the entropy of images being improved, problems arose: overall dark color tone, insufficient contrast and loss of image details.

Figure 5b shows the processed image using the dark channel prior algorithm: nearly all noise outside the blade in the image was eliminated. However, the top and bottom edge image details of the blade were severely lost, and the overall grayscale of the image was low. This result indicated that the details of the processed THz image of the target object would be lost using dark channel prior algorithm.

This being the case, we turned our attention to image enhancement algorithms based on retinex theory. For general image linear and nonlinear transformations, as well as image smoothing, image filtering and other image processing methods, only certain types of image features (such as brightness, saturation, contrast, etc.) can be enhanced. The image enhancement algorithm based on retinex theory can transform images by achieving a balance between various feature quantities. The method for different types of images can be optimized [43]. It has higher universality in practical application.

However, the performance of the single-scale retinex algorithm is not satisfactory in practical applications. The image processing using this method shows color distortion, excessive enhancement of boundaries and no significant improvement in detail information in areas with intense lighting. The multi-scale retinex algorithm is proposed by Jobson et al. to address these issues [44]. In essence, the multi-scale retinex algorithm is equivalent to performing multiple single-scale retinex algorithm treatments. Specifically, the image to be processed is processed using Gaussian filtering at multiple scales, and then the filtering results at each scale are weighted to obtain the estimated illumination component image of the required ambient light.

Figure 5c shows the result of image enhancement based on multi-scale retinex theory. On the one hand, the edge information of the blade in the image is well preserved and basically conforms to the shape of the object. On the other hand, the noise around the blade has also been enhanced. This noise could seriously affect the image quality. During the safety inspection process, it may interfere with the judgment of carried items.

To overcome the above problems, some scholars proposed using bilateral filtering functions instead of Gaussian functions as the surround function for the multi-scale retinex image enhancement algorithm [45]. The results showed that the algorithm’s performance was greatly improved, so it could simultaneously preserve the edge details of the image without halo phenomenon. However, this method requires a huge amount of computation and is time consuming; although it has excellent performance, the whole process of image processing is slow. 

In the classic dark channel algorithm, the brightest pixel value in the image is directly used to estimate the light value. Although it is simple and easy to operate, its results are easily affected by light-colored objects in the image, and the processed image is prone to the halo phenomenon. We solved this problem by narrowing the range of light values. The grayscale opening operation was performed on the processed image to eliminate the influence of light-colored objects, and then a specific estimation of the light value was made based on the specific situation of the image. By limiting the range of this interval, the occurrence of the halo phenomenon in the processing results was reduced.

The algorithm in this article uses the guided filtering function as the surround function of the multi-scale retinex image enhancement algorithm. Compared with the bilateral filtering function, the computational and time complexity of the guided filtering function was relatively low [46]. Moreover, in terms of preserving the edge details of the image, the effectiveness of the guided filtering function was significantly better than that of the bilateral filtering function. Therefore, guided filtering instead of Gaussian filtering was used in the improved algorithm. To avoid multiplication operations with high computational complexity during the operation process, the expression is usually converted to the logarithmic domain for calculation, which can greatly reduce the complexity of the algorithm. The processing formula can be expressed as:

(2) $$\begin{array}{ll} t_{i}\left(x,y\right)=\log\left(T_{i}\left(x,y\right)\right) & =\sum_{k=1}^{N}\omega_{k}\left(\log\left(I_{i}\left(x,y\right)\right)-\log\left(L_{i}\left(x,y\right)\right)\right)\\ & =\sum_{k=1}^{N}\omega_{k}\left(\log\left(I_{i}\left(x,y\right)\right)-\log\left(I_{i}\left(x,y\right)*f_{k}\left(x,y\right)\right)\right) \end{array},$$

here, *T_i_* (*x*, *y*) is the transmission component of the THz source after algorithm processing, *I_i_* (*x*, *y*) is the foggy image component of the image to be processed in the *i*-th color channel, and *L_i_* (*x*, *y*) is the illuminance component of the ambient light of the image to be processed in the *i*-th color channel, *ω_k_* is the weight coefficient of the *k*-th color channel during weighted processing, and *N* is the number of color channels in the image to be processed, which is the number of scale parameters (when *N* = 1, the image to be processed is a grayscale image; when *N* > 1, the image to be processed is a color image), *f_k_*(*x*, *y*) is the guided filtering function, and the expression of the guided filtering function can be written as:

(3) $$q_{i}=a_{k}I_{i}+b_{k},\forall i\in \Omega_{k}$$

where *q_i_* denotes the pixel value of the output image, *I*_i_ represents the pixel value of the guided image, *a_k_* and *b_k_* represents the invariant coefficient of the guided function when the center of the guided image is located in *k*-th pixel, and Ω*_k_* represents a square window, which is the processing range of the local processing process. The constant coefficients *a_k_* and *b_k_* can be calculated using the least squares method:

(4) $$a_{k}=\frac{\frac{1}{N_{w_{i}}}\sum_{i\in w_{i}}I_{i}p_{i}-\mu_{k}{\bar{p}}_{k}}{\sigma_{k}^{2}+\varepsilon }$$

(5) $$b_{k}={\bar{p}}_{k}-a_{k}\mu_{k}$$

where *w_i_*, *N_wi_*, *μ_k_*, *p_i_*, $\bar{p_{k}}$, $\sigma_{k}^{2}$ and *ε* denote the local window used, the number of pixels in the window used, the average value in the local window *w_i_*, the pixel value of the *i*-th pixel in the image to be processed, the mean in window *w_i_* in the image to be processed, the variance in the local window *w_i_* and an adjustment error parameter, respectively. 

The above operation is only a local processing of the image. Then, apply the above window operation to the entire image, and take the average to obtain the guided filtering function:

(6) $$f\left(I\right)=\frac{1}{N_{w_{i}}}\sum_{k,i\in w_{i}}\left(a_{k}I_{i}+b_{k}\right)$$

By substituting Equation (6) into (2), one can ultimately obtain the transmission component of the THz source carrying image details.

The result of image optimization using the image enhancement algorithm based on the improved DCP theory and the improved MSR theory is shown in Figure 5d. It shows a significant improvement in image clarity. The area of sample to be imaged is brighter. The edge detail information of the processed image is also well preserved, with no obvious loss of details, and the edges of the blade appear clearer. There are no obvious halos or distortions in the processed image. The THz image conforms to the shape of the actual object. Our algorithm not only optimized the edge information of the blade, but also almost eliminated the effect of noise around the object in the image completely. The comparison of these three images proves the optimization effect of the algorithm on THz images.

In addition, we compared our algorithm with the classic image enhancement algorithms DCP and MSR. Although the image processed by MSR algorithm exhibited the best enhancement on the target object, it had the most obvious foggy noise, which can affect the judgment of the shape of the imaging object. The image processed by the DCP algorithm had the least foggy noise, but it enhanced the object unevenly, with the central part of the blade having a darker color and the upper and lower ends having lighter color. Considering the balance between image enhancement and denoising effects, the algorithm proposed in this paper had the best optimization effect on THz images among these three algorithms.

Moreover, we evaluated the processing results of the image enhancement algorithm based on the improved DCP theory and the improved MSR theory from objective data indicators. Table 1 presents the objective data indicators, compared with the classic enhancement algorithms DCP and MSR, in terms of image brightness, contrast, entropy and peak signal-to-noise ratio (PSNR). Although the DCP algorithm exhibited good denoising performance, it sacrificed brightness, contrast and entropy. Even though the MSR algorithm significantly improved image brightness, contrast and information entropy, its peak signal-to-noise ratio was very low, which means that the image was severely distorted compared to the original image. This image distortion manifested as noise amplification in the processed image. Compared with the above two algorithms, our algorithm significantly improved the brightness, contrast and entropy of the image while maintaining a good PSNR.

## 4. Discussion

In the case of direct THz imaging with THz camera, the overall speed of imaging depends on not only the repetition rate of the THz source but also the frame rate of the THz camera, while the maximum size of the THz image depends on the size of THz beam and the intensity of the THz beam. The image resolution depends on both pixel size and the wavelength of the THz wave. In our experiment, an intense broadband THz source (generated from laser-plasmas) with a beam size of 76.2 mm and repetition rate of 1 kHz, as well as a central wavelength of ~25 μm was used for single-shot fast THz imaging. The intense THz wave allowed for beam expansion to expand the imaging area while maintaining sufficient THz intensity for imaging. The broadband THz wave weakened the effect of THz wave coherence in imaging results. Limited by the maximum frame rate of the THz camera, our imaging system worked at 8.5 fps for a maximum THz image size of ~7 cm. The average resolution was estimated as 146.2 μm. Our imaging results indicated that a clear THz image with its easily distinguished size and shape could be routinely obtained. This work improved the THz transmission imaging resolution and imaging size that depend on the THz wavelength and its intensity. Furthermore, a fast THz imaging for much larger target object could be developed based on this single-shot THz imaging system by scanning the large target objects with its 76.2 mm beam one by one. Compared with point scanning and line scanning THz imaging, this method can save much time. 

**Figure 6:**
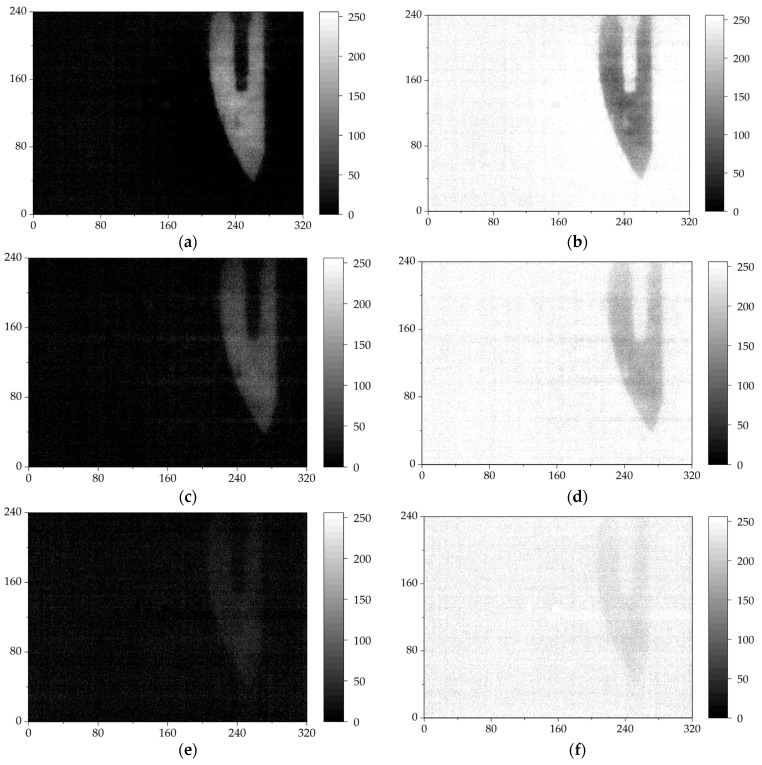
The imaging results of THz pulses with repetition rates of 1000 Hz, 333 Hz and 100 Hz (**a**,**c**,**e**) and the inversion of the images (**b**,**d**,**f**), respectively.

Furthermore, we tested the quality of THz transmission images at different repetition rates of THz source. If the repetition rate was decreased, each frame of the THz camera caught fewer THz pulses, which affected the signal-to-noise ratio (SNR) of the image. The results are shown in Figure 6. Figure 6a,c,e show images after subtracting the background image. Figure 6b,d,f are the inversion images of Figure 6a,c,e, respectively. Figure 6a,b show the THz transmission images of THz pulses at a repetition rate of 1 kHz. One can see that the blade in the THz image is very clear. When the repetition rate of THz pulses was reduced to 333 Hz, the THz transmission images were still relatively clear, as shown in Figure 6c,d. The details of the blade still can be easily distinguished. If the repetition rate of THz pulses was decreased to 100 Hz, the THz transmission images tended to become blurry and could be roughly identified, indicating a low SNR, as shown in Figure 6e,f.

This system also has the potential to achieve real-time imaging. If we replaced the THz camera with another with a frame rate greater than 24 fps, a real-time THz transmission imaging system could be realized in principle.

## 5. Conclusions

We developed a fast THz imaging system based on the single-shot technique in which an intense broadband THz source from laser–plasma interaction and a THz camera are used. With this THz imaging system, a single-shot THz image for a target object with a size of more than 7 cm and average resolution of 146.2 μm with a maximum frame rate of 8.5 fps can be routinely obtained, showing a potential application for fast THz imaging. By replacing detection devices with shorter response times, the THz imaging system acquisition speed realized real-time imaging in principle. Based on the imaging characteristics of the system, we propose an image enhancement algorithm based on the improved DCP theory and improved multi-scale retinex theory to enhance and optimize the THz imaging results. The brightness, contrast, entropy and PSNR of THz image were significantly improved with these algorithms. It is anticipated that an upgraded version of our fast THz imaging setup could be used in many fields.

## Figures and Tables

**Table 1:** Comparison between the algorithm in this article and classical algorithms.

Algorithm	Brightness	Contrast	Entropy	PSNR
Original image	16.97	148.77	3.48	/
DCP	9.06	63.01	2.95	23.50
MSR	30.28	586.94	4.02	21.61
Improved algorithm	23.54	310.32	3.53	27.25

## Data Availability

Data underlying the results presented in this paper are not publicly available at this time but may be obtained from the corresponding author upon reasonable request.

## Abbreviations

The following abbreviations are used in this manuscript:

THz	terahertz
fps	frames per second
DCP	dark channel prior
MSR	multi-scale retinex
PSNR	peak signal-to-noise ratio
